# Reducing transmission of SARS-CoV-2 with intranasal prophylaxis

**DOI:** 10.1016/j.ebiom.2020.103170

**Published:** 2020-12-16

**Authors:** Federico Boiardi, Justin Stebbing

**Affiliations:** a Colegio Internacional SEK-El Castillo, Madrid, Spain; b Department of Surgery and Cancer, Imperial College, London, UK

In a global effort to combat the COVID-19 pandemic, a diverse number of strategies may reduce viral transmission. Non-pharmaceutical interventions (*NPIs*) slow down epidemic spread without necessarily stopping it. In the absence of a distributed vaccine or highly effective antiviral, NPIs form one of the few readily available tactics we can employ to delay and/or reduce the spread. NPIs, however, have no influence on one's immunity to SARS-CoV-2, which is attained in one of two ways – infection, or immunisation. As SARS-CoV-2 infection probably does not result in lifelong immunity, the necessity for a therapy that might prevent transmission is of importance.

Both Regeneron's and Lilly's antibodies have recently received emergency use authorisation from the FDA as a treatment of mild-to-moderate COVID-19 [1]. Despite their promising results, the approach taken to derive such treatment comes with various impediments. Notably, the complexity and time associated with the production of monoclonal antibodies are likely to incur substantial costs. The necessity for infusion also poses greater limitations than would the opportunity to self-administer. It is therefore worthwhile to explore alternative methods for developing prophylaxis for COVID-19.

Such an alternative could include regulating transmission of SARS-CoV-2 by preventing attachment in the upper respiratory tract (*URT*). Previously, a group achieved this in animal models by administering the toll-like receptor-2 (*TLR-2*) agonist Pam2Cys in mice, in which reduced influenza virus levels in the URT and lungs were observed [2]. Pam2Cys seemed a suitable candidate for the treatment of other respiratory pathogens. However, the presence of oligo-lysine sequences in the compound's solubilising agent has been found to interfere with infection processes independent of TLR activation [3]. Specifically, they are known to enhance respiratory syncytial virus infection in primary epithelial, myeloid, and lymphoid cells. This effect was mitigated by incorporating polyethylene glycol as a solubilising agent, forming the INNA-X series of compounds.

In a study recently published in *EBioMedicine*, INNA-051, a similar compound, has been shown to potentially serve as a suitable intranasal (*i.n.*) prophylaxis for SARS-CoV-2 in a ferret challenge model [4]. INNA-051 is a synthetic antiviral that stimulates the innate immune system, also by binding to TLR-2 on the mucosa of airway epithelial cells. In the study, 24 female ferrets were split into 4 groups of 6. Groups 1 and 2 received different i.n. doses of INNA-051, group 3 received a mixed i.n. dose of INNA-051 and phosphate-buffered saline (*PBS*), and group 4 (the control group) received i.n. PBS 4 days prior to an i.n. SARS-CoV-2 challenge. The ferrets were then inoculated with SARS-CoV-2. No adverse reactions were seen during this period. At 5 days post-challenge (*pc*), all INNA-051-treated groups had reduced viral RNA levels (>10-fold in comparison to the control group). Beyond 10 days pc, levels of viral RNA were below the limit of quantification in all treatment groups. INNA-051-treated groups were combined into a single data set and then compared to the control group. Group 2, having received two 1 mL doses at 20 μg/mL (low dose), demonstrated the most favourable results. There was a 96% and 93% virus reduction in throat swabs and nasal washes, respectively, compared to untreated ferrets (*P* *<* *0.0001 and P* *=* *0.0107*).

Such positive results in a ferret model may be indicative of the antiviral's potential success in human trials. Like humans, ferrets express the metallopeptidase entry receptor for the virus, angiotensin-converting enzyme 2 (*ACE2*). These findings exemplify an encouraging potential for intranasal treatments to combat transmission and initial infection of SARS-CoV-2. Such an advance would be most beneficial for those that have an elevated risk of acquiring infection, such as elderly residents living in close communities, ie, those who are both susceptible and vulnerable.

Another study has taken a similar approach that instead directly targets the virus and prevents membrane fusion between itself and host cells from occurring [5]. The dimeric form of a SARS-CoV-2-derived lipopeptide [SARS-CoV-2-HRC-peg4]2-chol (*SARS-HP*) intervenes by disrupting structural rearrangements of spike glycoproteins that are essential for fusion. Twelve ferrets in total were included in the study, six of which receiving i.n. SARS-HP, and the others i.n. placebo. Of the ferrets given placebo, all were infected with the virus when co-housed for 24 h with directly inoculated donor ferrets. In contrast, of those that received SARS-HP, none contracted the virus. Additionally, variants of SARS-CoV-2, arising from the mutation of spike glycoproteins, as well as SARS-CoV and MERS-CoV were tested. SARS-HP completely inhibited the fusion of four SARS-CoV-2 strains, and showed considerable and partial effectiveness against SARS-CoV and MERS-CoV, respectively.

In both of these studies, advantages in comparison to alternatives such as immunisation may include fast protective response, the option of self-administration, inexpensive cost of production and distribution, and lack of systemic toxicity. Moreover, both demonstrate potential broad-spectrum usage across multiple respiratory viruses, as opposed to being targeted to a single CoV. For future development within the field, it should be noted that ferret ACE2, although expressed as efficiently as a SARS-CoV receptor to its human counterpart [6], binds to SARS-CoV-2 inefficiently [7]. Other *in vivo* models, such as hamsters [7], may further delineate protective correlates in humans. However, there remains the need for human trials, which should ideally be randomised [8].

At the very least, the notion of intranasal prophylaxis has exhibited the potential to open doors to promising innovative paths for forthcoming investigation.

## Declaration of Interests

JS conflicts can be found at: https://www.nature.com/onc/editors

None are relevant here.

## Contributors

The authors confirm sole responsibility for the conception and preparation of this invited Commentary.
